# Effects of a monophasic combined oral contraceptive containing nomegestrol acetate and 17β-oestradiol in comparison to one containing levonorgestrel and ethinylestradiol on markers of endocrine function

**DOI:** 10.3109/13625187.2011.614363

**Published:** 2011-09-26

**Authors:** Ulla M Ågren, Marjatta Anttilat, Kristiina Mäenpää-Liukko, Maija-Liisa Rantala, Hilkka Rautiainen, Werner F Sommer, Ellen Mommers

**Affiliations:** * FSHS Kuopio, Kuopio, Finland and MSD, Espoo, Finland; † FSHS Tampere, Tampere, Finland; ‡ Turun Gynekologikeskus Ky, Turku, Finland; § Lääkärikeskus Adenova, Espoo, Finland; # Lääkärikeskus Gyneko Oy, Oulu, Finland; ^ MSD, Oss, The Netherlands

**Keywords:** Oral contraceptives, Nomegestrol acetate, 17β-oestradiol, Adrenal, Thyroid, Androgen, SHBG

## Abstract

**Objectives:**

To compare the effects of two monophasic combined oral contraceptives, containing either nomegestrol acetate/17β-oestradiol (NOMAC/E2) or levonorgestrel/ ethinylestradiol (LNG/EE) on endocrine function, androgens, and sex hormone-binding globulin (SHBG).

**Methods:**

Randomised, open-label, multi-centre trial involving 121 healthy women, aged 18-50 years old. Participants received NOMAC/E2 (2.5 mg/1.5 mg) in a 24/4-day regimen (*n* = 60) or LNG/EE (150 μg/30 μg) in a 21/7-day regimen (*n* = 61) for six cycles. The primary outcome was the change from baseline to cycle 6 in markers of adrenal and thyroid function, androgens, and SHBG.

**Results:**

Total cortisol, corticosteroid-binding globulin (CBG), and thyroxine-binding globulin (TBG) increased from baseline in both groups, with significantly greater increases in the LNG/EE group. No relevant changes from baseline or differences between the groups were observed for thyroid-stimulating hormone (TSH) and free thyroxine (T_4_). Androgens and androgen precursors decreased from baseline in both groups, with significantly greater decreases in the LNG/EE group (except for free testosterone). A greater increase in SHBG was observed with NOMAC/E2 than with LNG/EE.

**Conclusions:**

NOMAC/E2 has significantly less influence on markers of adrenal and thyroid function and androgens than LNG/EE. The clinical relevance of these findings requires further study.

## INTRODUCTION

Combined oral contraceptives (COCs) are the most popular form of reversible contraception in the world^1^. As COCs have evolved, the two most important formulation changes consisted of a reduction in the dose of the oestrogenic component ethinylestradiol (EE) from ≥ 50 μg to < 35 μg per pill, and the introduction of newer progestogens. Together, these changes have helped to reduce cardiovascular events,and have improved the safety and tolerability profiles of COCs^2^,^3^.

The incorporation of oestrogens and progestogens that are structurally similar or identical to endogenous hormones represent the most recent change in the evolution of COCs^4^,^5^. In particular, 17β-oestradiol (E2) has been investigated as an alternative to synthetic EE. Early attempts to incorporate E2 into COCs were unsuccessful due to poor cycle control, especially when administered as part of a monophasic or biphasic regimen^6^-^9^. Despite these early setbacks, it is still hypothesised that the use of E2 in combination with a suitable progestogen would improve the safety of COCs and patient tolerability^10^.

Nomegestrol acetate (NOMAC) is a progestogen structurally similar to progesterone that is devoid of oestrogenic, androgenic, glucocorticoid, and mineralocorticoid activity but it exerts antioestrogenic effects on the endometrium and has a moderate antiandrogenic activity^11^. The combination of NOMAC with E2 (NOMAC/E2) was developed as a monophasic COC. Previous studies have shown that NOMAC/E2 administered in a 24/4-day dosing regimen had suppressive effects on the ovaries, cervical mucus and endometrium that were at least as strong as those of a comparator COC containing drospirenone and EE^12^.

Several studies have shown that COCs can affect adrenal and thyroid function as well as androgen levels^13^-^15^. The objective of this six-cycle study was to determine the effects of NOMAC/E2 on surrogate markers of adrenal and thyroid function, androgens, androgen precursors, and sex hormone-binding globulin (SHBG) in comparison to the COC levonorgestrel (LNG)/EE (150 μg/30 μg). The effects of NOMAC/E2 on haemostasis, lipids, and carbohydrate metabolism were also assessed in this trial and are reported elsewhere^16^.

## METHODS

This study was a randomised, open-label, comparative, parallel-design clinical study. Five centres in Finland participated in the trial. Advertisements were used to recruit women from the regions surrounding the study centres (Kuopio, Tampere, Turku, Espoo, and Oulu). Throughout the trial, study volunteers were compensated for their time, travel costs, and inconvenience associated with study visits.

The study was conducted in compliance with the ethical principles described in the Declaration of Helsinki and the International Conference on Harmonisation (ICH) guideline for Good Clinical Practice (GCP). At each study centre, the Independent Ethics Committee reviewed and approved the trial protocol (NCT00511355). Prior to treatment, written informed consent was obtained from all study participants.

### Participants

Healthy, sexually active women aged 18-50 years with a body mass index (BMI) between 17 and 29 kg/m were eligible for the study if they had no contraindications for the use of contraceptive steroids and had not taken any other hormonal treatment (except contraceptives) within two months prior to screening. The contraindications for contraceptives were in line with the World Health Organisation's medical eligibility criteria for contraceptive use^17^. Additionally, women were excluded from the study if they had an abnormal cervical smear or a clinically relevant abnormal laboratory finding, were breastfeeding, or if they used liver-enzyme-inducing drugs, investigational drugs, or pharmacological agents that can affect haemostasis (e.g., vitamin K, non-steroidal anti-inflammatory drugs, including aspirin).

### Treatment and study design

The objectives of this trial were to assess and compare the effects of NOMAC/E2 and LNG/EE on surrogate markers of adrenal and thyroid function, androgens, androgen precursors, and SHBG levels.

The trial consisted of one pretreatment cycle (cycle 0), followed by six 28-day treatment cycles (cycles 1-6) and a post-treatment visit. All study visits in which blood samples needed to be taken took place in the morning, when participants were fasting. At the screening visit, eligibility for participation was determined and baseline data were collected. Women were instructed to discontinue the use of hormonal contraceptives and to use condoms whenever necessary. The first menstruation after the screening visit was considered a withdrawal bleeding, and the pretreatment cycle started during the next ('spontaneous') menstrual period. The pretreatment cycle visit occurred during the second half of this cycle. During this visit, blood was taken for baseline assessment of markers of adrenal and thyroid function, androgens, androgen precursors, and SHBG. This would ensure a period of at least six weeks between last contraceptive use and baseline measurements (i.e., four weeks until spontaneous menstruation, plus two weeks until baseline assessment).

Women who were eligible for enrolement were randomised in a 1:1 ratio to receive daily either a COC containing 2.5 mg NOMAC and 1.5 mg E2, or the comparator COC containing 150 μg LNG and 30 μg EE. Randomisation was performed using blocks with randomly permuted block sizes and an interactive voice response system. Due to the broadened age range compared with previous trials with COCs, women were stratified into two age groups (18-35 years old and 36-50 years old) to ensure that approximately 20% of the participants were between 36—50 years of age at screening. For six consecutive 28-day cycles, women took one oral tablet of study medication at approximately the same time each day (day 1 to day 28). The NOMAC/E2 regimen consisted of 24 days of active pills followed by four days of placebo pills; the LNG/EE regimen consisted of 21 days of active pills followed by seven days of placebo pills. Between days 15 and 21 of treatment cycles 3 and 6, participants returned to the clinic in a fasted state and blood was collected. A final visit was scheduled between 8 and 14 days after the last tablet was taken in cycle 6 or after early discontinuation of treatment for the gathering of general follow-up data.

### Laboratory measurements

All laboratory examinations were performed by the Bio Analytical Research Corporation (BARC) in Gent, Belgium. A manual with complete instructions on blood sampling, processing, storage, and shipment was provided to the centres by the central laboratory (BARC).

#### Surrogate markers of adrenal and thyroid function

Electrochemiluminescence immunoassays (ECLIAs; Roche Diagnostics) were used to measure serum levels of total cortisol, thyroid-stimulating hormone (TSH), and free thyroxine (T_4_). Radioimmunoassays (RIAs) were used to measure corticosteroid-binding globulin (CBG; Biosource) and thyroxine-binding globulin (TBG; Brahms).

#### Androgens and androgen precursors

Total testosterone was measured by ECLIA (Roche Diagnostics). Dehydroepiandrosterone sulphate (DHEAS), androstenedione, and dihydrotestosterone (DHT) were measured by radioimmunoassay (Immunotech; DPC Coat A Count, and DPC, respectively). Levels of free testosterone were calculated from total testosterone and SHBG as described by Vermeulen *et al*. 18.

#### SHBG

Serum SHBG was measured by ECLIA (Roche Diagnostics).

### Statistical analysis

The trial was set up to confirm the expected differences in changes from baseline between the treatment groups in several metabolic indices, especially prothrombin fragment 1+2, D-dimer, and antithrombin III, as reported elsewhere^16^. In short, based on expected effect sizes (i.e., differences between the treatment groups divided by the standard deviation) of 0.6 or higher observed in previous trials, a sample size of 42 evaluable participants per treatment group was needed using a two-sided statistical test with 80% power and a significance level of 5%. This power to detect moderately large effect sizes of 0.6 was also considered appropriate for surrogate markers of adrenal and thyroid function, androgens, and androgen precursors assessed in the trial. Compensating for up to 20% premature discontinuations from treatment and accounting for a potential power loss due to the non-parametric analysis, in total 60 women were to be randomised per treatment group.

The analyses of the cycle 6 assessments were considered the primary outcome, whereas the analyses of the cycle 3 assessments were regarded as secondary outcomes. Analyses were performed for all participants who had taken at least one tablet of either study medication. Summary statistics are presented as mean values with standard deviations (SDs) and median values with interquartile ranges (IQRs). Data are presented for baseline (defined as the last measurement before administration of the first study medication) and treatment cycles 3 and 6; changes from baseline to cycle 6 were calculated per index assessed. All tests of statistical significance were performed at the 5% error level. The Cochran-Mantel-Haenszel test, 95% confidence intervals (CIs; based on the Hodges-Lehmann approach^19^), frequency tables, and descriptive statistics were used for data analyses. No correction for multiplicity was applied.

## RESULTS

**Figure 1:**
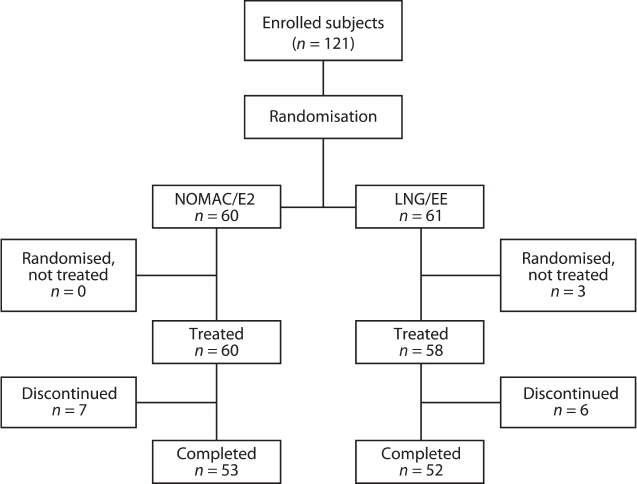
Flow chart of participants. NOMAC/E2, nomegestrol acetate/17β-oestradiol; LNG/EE, levonorgestrel/ ethinylestradiol.

A total of 121 women were randomised to receive either NOMAC/E2 or LNG/EE (Figure 1). All women in the NOMAC/E2 group (*n* = 60) received treatment, whereas three of the women in the LNG/EE group (*n* = 61) did not because of a pretreatment adverse event (AE) ('acne'), withdrawn consent, or other personal reason (found a new job). Seven women (11.7%) in the NOMAC/E2 group and six women (10.3%) in the LNG/EE group discontinued treatment before the end of the trial. Of these 13 discontinuers, eight women (four in each group) discontinued treatment due to an AE, one due to pregnancy wish, one moved to another city, and three women were lost to follow-up. Overall tablet intake compliance was high in both treatment groups: 93.1% and 87.7% of women in the NOMAC/ E2 and LNG/EE groups, respectively, took the daily tablet on at least 95% of treatment days.

The demographic and clinical characteristics were similar between the two treatment groups at baseline (Table 1), with the exception of smoking prevalence, which was higher among women in the NOMAC/ E2 group. The majority of smokers were light smokers (15.0% and 8.6% of all NOMAC/E2 and LNG/ EE users, respectively), consuming on average less than 10 cigarettes per day. Baseline values for all measured indices were balanced between the two treatment groups (Tables 2-4). The mean age was 28.7 years. All but one of the participants (99.2%) were White. The effects on surrogate markers of adrenal and thyroid function, androgens, androgen precursors, and SHBG were similar for the two age groups (18-35 years old and 36-50 years old). The results are therefore presented only for the age groups combined.

**Table 1:** Summary of participants’ characteristics at screening.

	*N0MAC/E2 (n = 60)*	*LNG/EE (n = 58)*	*Total (n = 118)*
Age (years), mean (SD)	28.2 (8.2)	29.1 (78)	28.7 (8.0)
Race, *n* (%)			
Black	1 (1.7)	0	1 (0.8)
White	59 (98.3)	58 (100.0)	117 (99.2)
Weight (kg), mean (SD)	62.8 (9.7)	61.7 (9.0)	62.3 (9.3)
BMI (kg/m^2^), mean (SD)	23.0 (2.9)	22.3 (2.5)	22.7 (2.7)
Parity, *n* (%)			
Never pregnant	35 (58.3)	34 (58.6)	69 (58.5)
No pregnancy ≥ 28 weeks	4 (6.7)	3 (5.2)	7 (5.9)
1	7 (11.7)	6 (10.3)	13 (11.0)
≥2	14 (23.3)	15 (25.9)	29 (24.6)
Last contraceptive method used within three months prior to screening*, *n* (%)			
None	1 (1.7)	4 (6.9)	5 (4.2)
Combined oral contraceptive	19 (31.7)	22 (37.9)	41 (34.7)
Progestogen-only-pill	3 (5.0)	1 (1.7)	4 (3.4)
ILJD (hormonal)	5 (8.3)	1 (1.7)	6(5.1)
ILJD (non-hormonal)	1 (1.7)	2 (3.4)	3 (2.5)
Vaginal ring or transdermal patch	5 (8.3)	7 (12.1)	12 (10.2)
Foam, condom, suppositories, diaphragm	26 (43.3)	21 (36.2)	47 (39.8)
Smoking status			
Smokers, *n* (%)	14 (23.3)	7 (12.1)	21 (17.8)
Non-smokers, *n* (%)	46 (76.7)	51 (87.9)	97 (82.2)

NOMAC/E2, nomegestrol acetate/17β-oestradiol; LNG/EE, levonorgestrel/ethinylestradiol; SD, standard deviation; BMI, body mass index; NA, not applicable; ILJD, intrauterine device.

* Most important contraceptive method.

**Table 2:** Effects of nomegestrol acetate/17β-oestradiol and levonorgestrel/ethinylestradiol on adrenal and thyroid indices.

		*Nomegestrol acetate/17β-oestradiol*				*Levonorgestrel/ethinylestradiol*			
			
*Index*	*Assessment*	*Mean*	*SD*	*Median*	*IQR*	*Mean*	*SD*	*Median*	*IQR*
Total	Baseline	482	129	469	166	502	153	524	166
cortisol	Cycle 3	610	186	607	221	975	215	1021	248
(nmol/l)	Cycle 6	608	167	580	193	944	183	952	207
	Change from baseline to cycle 6	123	195	111	166	432	172	441	193
	% change from baseline to cycle 6	32.9	45.3	24.9	39.8	97.4	65.2	82.2	52.8
CBG (nmol/l)	Baseline	910	201	867	232	932	163	917	213
	Cycle 3	1182	367	1091	326	2086	430	2069	588
	Cycle 6	1116	253	1053	253	1980	389	1929	536
	Change from baseline to cycle 6	220	264	194	225	1047	402	967	523
	% change from baseline to cycle 6	27.4	29.2	21.9	28.9	117.7	53.4	111.0	81.0
TSH (mU/l)	Baseline	2.69	1.28	2.38	1.61	2.20	1.08	1.92	1.12
	Cycle 3	3.03	1.52	2.71	1.77	3.78	10.23	2.52	1.61
	Cycle 6	2.96	2.05	2.56	1.70	2.75	3.36	2.19	0.96
	Change from baseline to cycle 6	0.25	1.75	−0.04	1.16	0.50	3.07	0.11	0.91
	% change from baseline to cycle 6	13.3	63.6	−2.2	50.6	22.8	78.5	6.9	49.7
Free T_4_ (pmol/l)	Baseline	14.0	1.6	14.0	1.9	14.1	1.5	14.0	2.2
	Cycle 3	15.5	1.9	15.2	3.0	15.7	2.1	15.8	2.9
	Cycle 6	15.9	2.0	15.8	2.5	15.7	2.1	15.3	3.3
	Change from baseline to cycle 6	1.8	1.8	1.7	2.7	1.4	1.7	1.3	2.1
	% change from baseline to cycle 6	13.4	13.8	11.8	19.1	10.2	11.9	9.5	14.2
TBG	Baseline (mg/l)	20.3	2.9	20.1	4.1	20.3	3.3	20.1	4.4
	Cycle 3	24.0	2.8	24.4	3.8	28.1	4.7	28.2	6.8
	Cycle 6	24.2	3.6	24.0	3.6	28.4	5.3	27.9	7.4
	Change from baseline to cycle 6	4.0	3.9	4.3	4.0	7.9	3.8	7.7	5.4
	% change from baseline to cycle 6	21.6	20.6	21.0	23.2	39.0	19.5	38.1	30.2

SD, standard deviation; IQR, interquartile range; CBG, corticosteroid-binding globulin;TSH, thyroid-stimulating hormone; T_4_, thyroxine; TBG, thyroxine-binding globulin.

### Analyses related to surrogate markers of adrenal and thyroid function

Total cortisol, CBG, andTBG concentrations increased from baseline to cycle 6 in both treatment groups, with a significantly more pronounced rise in the LNG/EE group (Tables 2 and 5; *p* < 0.001). For TSH and free T_4_, changes from baseline to cycle 6 were small, with no statistically significant differences between NOMAC/E2 and LNG/EE. For all adrenal and thyroid indices, changes from baseline to cycle 3 were similar to changes from baseline to cycle 6.

### Androgens, androgen precursors, and SHBG

For androgens and androgen precursors, a decrease from baseline to cycle 6 was observed, which was greater in the LNG/EE group than in the NOMAC/ E2 group (Table 3). The differences between the treatment groups in change from baseline to cycle 6 were statistically significant for all androgens and androgen precursors (Table 5; *p* < 0.05) except for free testosterone. Both treatments were associated with increases in median SHBG concentrations (Table 4), with a significantly greater increase in the NOMAC/E2 group (44%) compared with the LNG/ EE group (22%) at cycle 6 (*p* = 0.019; Table 5).

**Table 3:** Effects of nomegestrol acetate/17β-oestradiol and levonorgestrel/ethinylestradiol on androgenic indices.

		*Nomegestrol acetate/17β-oestradiol*				*Levonorgestrel/ethinylestradiol*			
			
*Index*	*Assessment*	*Mean*	*SD*	*Median*	*IQR*	*Mean*	*SD*	*Median*	*IQR*
Total testosterone (nmol/l)	Baseline	1.68	0.75	1.58	1.13	1.90	0.94	1.82	0.97
	Cycle 3	1.12	0.60	1.04	0.77	0.91	0.56	0.89	0.80
	Cycle 6	1.23	0.86	1.04	0.77	0.91	0.58	0.80	0.71
	Change from baseline to cycle 6	−0.47	0.83	−0.46	0.79	−1.02	0.72	−0.88	0.81
	% change from baseline to cycle 6	−25.2	38.1	−31.1	42.8	−53.8	25.4	−60.2	29.2
Free testosterone (pmol/l)	Baseline	24.5	14.9	20.8	13.9	26.3	16.6	24.3	17.3
	Cycle 3	12.3	6.4	10.4	10.5	10.0	6.7	10.4	10.4
	Cycle 6	12.8	8.8	10.4	7.0	9.9	6.7	6.9	10.4
	Change from baseline to cycle 6	−12.2	13.6	−10.4	10.4	−16.6	14.0	−13.9	15.7
	% change from baseline to cycle 6	−41.5	39.4	−50.4	33.5	−57.8	29.4	−66.5	25.0
DHEAS (μmol/I)	Baseline	4.9	2.2	4.6	2.6	5.2	2.3	5.0	3.0
	Cycle 3	4.5	2.0	4.3	2.7	4.3	2.1	4.0	2.7
	Cycle 6	4.3	1.8	4.2	1.8	4.0	1.9	3.5	2.2
	Change from baseline to cycle 6	−0.7	1.6	−0.7	1.3	−1.3	1.2	−1.1	1.2
	% change from baseline to cycle 6	−10.1	23.9	−14.0	27.9	−22.9	18.0	−24.8	15.9
Androstenedione (nmol/l)	Baseline	9.6	3.5	9.0	4.1	10.3	3.9	9.4	4.4
	Cycle 3	7.6	2.5	7.5	2.8	6.5	2.7	6.3	3.3
	Cycle 6	8.2	3.0	8.1	3.6	7.0	3.4	6.3	4.5
	Change from baseline to cycle 6	−1.7	3.0	−1.4	3.6	−3.6	3.2	−4.0	3.7
	% change from baseline to cycle 6	−13.2	30.2	−15.5	37.3	−33.0	27.3	−36.4	33.3
DHT (nmol/l)	Baseline	0.59	0.21	0.55	0.23	0.62	0.26	0.52	0.34
	Cycle 3	0.61	0.25	0.58	0.27	0.47	0.21	0.47	0.31
	Cycle 6	0.53	0.28	0.48	0.38	0.36	0.19	0.33	0.26
	Change from baseline to cycle 6	−0.08	0.30	−0.14	0.41	−0.28	0.26	−0.28	0.34
	% change from baseline to cycle 6	−73	47.7	−22.6	71.8	−38.1	37.8	−48.3	38.4

SD, standard deviation; IQR, interquartile range; DHEAS, dehydroepiandrosterone sulphate; DHT dihydrotestosterone.

**Table 4:** Effects of nomegestrol acetate/17β-oestradiol and levonorgestrel/ethinylestradiol on SHBG.

		*Nomegestrol acetate/17β-oestradiol*				*Levonorgestrel/ethinylestradiol*			
			
*Index*	*Assessment*	*Mean*	*SD*	*Median*	*IQR*	*Mean*	*SD*	*Median*	*IQR*
SHBG (nmol/l)	Baseline	73.5	34.3	65.5	33.6	775	26.2	76.5	40.6
	Cycle 3	101.4	37.1	96.3	55.5	103.7	35.5	93.9	47.6
	Cycle 6	108.2	43.6	107.9	57.8	99.8	31.5	98.3	30.8
	Change from baseline to cycle 6	35.3	41.4	33.8	52.0	20.7	30.4	15.3	42.6
	% change from baseline to cycle 6	61.7	67.0	44.1	78.8	36.4	53.3	22.4	64.1

SD, standard deviation; IQR, interquartile range; SHBG, sex hormone-binding globulin.

**Table 5:** Differences between treatment groups (nomegestrol acetate/17β-oestradiol versus levonorgestrel/ ethinylestradiol) in median absolute changes from baseline to cycle 6 for adrenal, thyroid, and androgenic indices.

	*Difference in change from baseline to cycle 6 NOMAC/E2 vs. LNG/EE*	
	
*Index*	Estimate (95% CD*	p-value**
*Adrenal and thyroid indices*		
Total cortisol (nmol/l)	−303 (−359;−248)	<0.001
CBG (nmol/l)	−811 (−945;−689)	<0.001
TSH (mU/l)	−0.10 (−0.46; 0.27)	0.57
Free T_4_ (pmol/l)	0.45 (−0.30; 1.10)	0.18
TBG (mg/l)	−3.60 (−5.20;−2.20)	<0.001
*Androgens and androgen precursors*		
Total testosterone (nmol/l)	0.49 (0.28; 0.69)	<0.001
Free testosterone (pmol/l)	3.50 (0.00; 700)	0.13
DHEAS (nmol/l)	0.63 (0.16; 1.07)	0.013
Androstenedione (nmol/l)	1.96 (0.87; 3.24)	<0.001
DHT (nmol/l)	0.20 (0.08; 0.31)	<0.001
*Other Index*		
SHBG (nmol/l)	15.6 (3.2, 28.8)	0.019

* Point estimates of the difference and two-sided 95% confidence intervals (CIs) by the Hodges-Lehmann approach.

** Differences between groups; Cochran-Mantel-Haenszel test adjusted for age class using standardised midranks, applied on changes from baseline.

NOMAC/E2, nomegestrol acetate/17β-oestradiol; LNG/EE, levonorgestrel/ethinylestradiol; CBG, corticosteroid-binding globulin; TSH, thyroid-stimulating hormone; T_4_, thyroxine; TBG, thyroxine-binding globulin; DHEAS, dehydroepiandrosterone sulphate; DHT dihydrotestosterone; SHBG, sex hormone-binding globulin.

For all androgens, androgen precursors, and SHBG, changes from baseline to cycle 3 were in general similar to changes from baseline to cycle 6.

### Contraceptive efficacy and tolerability

No pregnancies occurred during the trial in either treatment group. NOMAC/E2 had a similar AE profile as LNG/EE, and both COCs were generally well tolerated. The most frequently reported AEs (for NOMAC/E2 and LNG/EE, respectively) were upper respiratory tract infection (six and five participants), headache (three and seven participants), and acne (two and four participants). One serious adverse event (SAE), the worsening of a congenital mitral valve leak, was reported in the NOMAC/E2 group. Immediately after clinical evaluation, the woman was withdrawn from the study. No serious AEs were reported in the LNG/EE group. Eight women (four women in each group) discontinued treatment during the study because of an AE. Reasons for discontinuation due to an AE included depression, nausea, and a combination of tachycardia, pain in the calf, and weakness in the limb (NOMAC/E2). In the LNG/EE group, AEs that led to discontinuation included decreased sexual desire, nausea, and headache.

## DISCUSSION

This randomised study compared the effects of NOMAC/E2 administered in a 24-day regimen with LNG/EE administered in a 21-day regimen on surrogate markers that are indicative of adrenal and thyroid function, androgens, and androgen precursors. It appears that NOMAC/E2 has less influence than LNG/EE on these indices.

While both treatments caused CBG and total cortisol levels to rise, increases were significantly greater in women who received LNG/EE. Elevated CBG, which is commonly observed with COC use, is related to an oestrogen-induced increase in the hepatic synthesis of serum proteins^13^,^20^-^21^. The effect of oestrogens on protein synthesis in the liver is not counteracted by progestogens^20^. Consequently, the significantly lesser increase in CBG observed in women taking NOMAC/E2 is likely the result of the substitution of EE with E2. This is in line with the findings of a previously performed study, which showed that the effect of 2 mg oestradiol valerate (which is equivalent to 1.52 mg E2^22^) on CBG was considerably less than that of 10 μg EE^15^. The elevations in total cortisol are probably caused by an increased concentration of CBG, resulting in de—creased clearance and not by a direct effect on adrenal function. This was also concluded in previous studies that assessed the effects of COC use on total cortisol^14^,^23^,^24^.

The increase in TBG as seen in the NOMAC/E2 group and to a greater extent in the LNG/EE group is also considered to be caused by the oestrogenic component of COCs^15^-^25^-^26^. As for CBG, the EE-induced, dose-dependent increase in TBG is generally not suppressed by progestogens, although a progestogen with androgenic activity may counteract this effect to some extent^15^. A rise in TBG results in the reduced clearance of tri-iodothyronine (T_3_) and T_4_, thus increasing total T_3_ and T_4_. COC use, however, appears to have little or no effect on physiologically active, free fractions of thyroid hormones^14^,^24^-^26^. Also, no significant changes in free T_4_ or in TSH were observed in either group after six months of treatment, which is in agreement with results from other studies^14^-^24^-^25^.

Both NOMAC/E2 and LNG/EE induced a drop in the concentrations of all androgens and androgen precursors, with far more pronounced decreases occurring in women receiving LNG/EE. In general, COCs suppress both ovarian and adrenal androgen synthesis, resulting in decreased levels of the androgen precursors androstenedione and DHEAS, as well as free and total testosterone^26^. The more pronounced drop in free testosterone relative to total testosterone in women receiving NOMAC/E2 is likely the result of the increase in SHBG, which was greater for NOMAC/E2 than for LNG/EE. LNG, a progestogen with residual androgenicity, counteracts the EE-induced increase in SHBG, while NOMAC, a non-androgenic progestogen, does not have this effect. While the suppression of androgenic activity may be a useful treatment in women with hyperan-drogenic symptoms like acne, it may potentially also adversely affect sexual function in women^27^-^29^.

The main limitation of this study is the use of surrogate endpoints. While the surrogate markers assessed in this study are indicative of adrenal and thyroid function, they do not directly assess endocrine function. In addition, the relevance of the drops in androgens and androgenic precursors on clinical endpoints like acne and sexual function cannot be determined in a relatively small trial like this. The significance of these findings can only be determined in large clinical studies with a sufficiently long duration of treatment.

Since we observed an imbalance in smokers between the treatment groups (23.3% vs. 12.1% in the NOMAC/ E2 and LNG/EE groups, respectively), we investigated this factor in more detail. Although smoking was shown to have a significant effect on total cortisol, androstenedione, DHT, and SHBG, with larger increases (or less pronounced decreases) in smokers in comparison to non-smokers (results not shown), analysis of variance methods based on ranks showed that all treatment effects adjusted for smoking were completely consistent with the results unadjusted for smoking.

In summary, this study demonstrated that the monophasic COC NOMAC/E2 has less influence on surrogate markers of adrenal and thyroid function, androgens, and androgen precursors than LNG/ EE. Large clinical or retrospective studies will be needed to determine whether these differences in surrogate endpoints have any clinical relevance.
